# De-escalation of elective radiotherapy guided by FDG-PET lowers modeled late swallowing-related toxicity in head and neck cancer

**DOI:** 10.1016/j.ctro.2026.101156

**Published:** 2026-04-01

**Authors:** Florian Stritzke, Katharina Dvornikovich, Hin Lau, Thomas Tessonnier, Philipp Schröter, David Neugebauer, Nils Netzer, Lukas Bauer, Rubens Thölken, Erik Winter, Semi Harrabi, Andrea Mairani, Uwe Haberkorn, Jürgen Debus, Thomas Held

**Affiliations:** a Heidelberg University, Heidelberg Faculty of Medicine, Heidelberg, Germany; b Department of Radiation Oncology, Heidelberg University Hospital, Heidelberg University, Heidelberg, Germany; c Heidelberg Institute of Radiation Oncology (HIRO), Heidelberg, Germany; d National Center for Tumor diseases (NCT), Heidelberg, Germany; e Clinical Cooperation Unit Radiation Oncology, German Cancer Research Center (DKFZ), Heidelberg, Germany; f Heidelberg Ion Beam Therapy Center (HIT), Heidelberg, Germany; g Department of Otorhinolaryngology, Head and Neck Surgery, Heidelberg University Hospital, Heidelberg University, Heidelberg, Germany; h Department of Nuclear Medicine, Heidelberg University Hospital, Heidelberg, Germany; i German Cancer Consortium (DKTK), partner site Heidelberg, German Cancer Research Center (DKFZ), Heidelberg, Germany

**Keywords:** Head and neck cancer, FDG-PET, Elective nodal irradiation, De-escalation, NTCP modeling, Dysphagia

## Abstract

**Purpose:**

Whether FDG-PET–guided de-escalation of elective target volumes in radiotherapy of the head and neck reduces late toxicity remains uncertain. Therefore, we compared predicted toxicity of two de-escalation strategies—reduced elective nodal irradiation (RNI) and involved node irradiation (INI)—with standard elective nodal irradiation (ENI).

**Materials and Methods:**

In a within-patient plan comparison (n = 26), we generated RNI and INI plans using the same FDG-PET informed high-/intermediate-risk targets as ENI. RNI limited elective volumes to within 2 cm cranio-caudal from gross disease; INI omitted elective treatment. Proton plans were created in representative oropharyngeal cases. Co-primary outcomes were six-month normal-tissue complication probabilities (NTCPs) for dysphagia, feeding-tube dependence, and xerostomia.

**Results:**

Median total planning target volume decreased from 540 cm^3^ (ENI) to 418 cm^3^ (RNI) and 173 cm^3^ (INI). RNI and INI reduced modeled dysphagia risk by 5.9 percentage points (pp) (95% CI 2.3 to 9.6 pp; p_adj_ = 0.005) and 11.0 pp (6.9 to 15.2 pp; p_adj_ < 0.001), feeding-tube dependence by 2.8 pp (1.2 to 4.5 pp; p_adj_ = 0.005) and 4.2 pp (2.5 to 6.0 pp; p_adj_ < 0.001), and xerostomia by 2.8 pp (0.0 to 5.7 pp; p_adj_ = 0.054) and 8.8 pp (5.5 to 12.2 pp; p_adj_ < 0.001), respectively. Predicted benefits were greatest in hypopharyngeal and laryngeal cancers. In selected cases, de-escalated proton therapy further reduced organ-at-risk dose.

**Conclusions:**

FDG-PET–guided volumetric de-escalation was associated with lower modeled late swallowing-related toxicity. These findings support the rationale for ongoing and future trials investigating volumetric de-escalation strategies.

## Highlights

• FDG-PET–guided volumetric de-escalation reduced modeled swallowing-related toxicity.
• Reduced elective and involved-node irradiation are predicted to reduce dysphagia.
• Hypopharyngeal and laryngeal cancers showed the greatest predicted benefit.
• Combining protons with de-escalation may further enhance organ-at-risk sparing.
• Effect estimates may inform patient selection for future de-escalation trials.

## Introduction

Head and neck cancer ranks among the most common malignancies worldwide, and radiotherapy is a cornerstone of its treatment. While offering curative potential, radiotherapy causes enduring side effects, including swallowing dysfunction. Health-related quality of life remains impaired up to five years after treatment [1], with about half of survivors expressing concerns about swallowing [2]. Dysphagia also increases mortality risk, with aspiration pneumonia accounting for approximately one in five non-cancer-related deaths [3].

Radiation dose parameters at pharyngeal constrictor muscles (PCM) and the supraglottic larynx strongly correlate with dysphagia [4], [5]. Similarly, other normal-tissue complication probabilities (NTCP), including xerostomia and tube feeding, are linked to dose distribution in tissues often located close to the radiation target [6], [7], [8], [9]. Enabling precise radiation delivery, IMRT reduces toxicity, and improves patients' quality of life compared to conventional radiotherapy [10]. Proton therapy can additionally spare significant radiation dose at critical structures like PCM [11]. However, large cervical target volumes might dilute the benefit of precision techniques compared to selective neck irradiation.

More selective target delineation is supported by the accurate identification of metastatic lymph nodes using FDG-PET/CT, which achieves a sensitivity of 83% and a specificity of 96% for detecting metastatic lymph node levels, compared to 63% sensitivity with conventional imaging [12]. The negative predictive value of FDG-negative nodal levels is 95% [13]. De-escalation trials requiring pretreatment FDG-PET have suggested that reducing elective nodal irradiation (ENI) might preserve regional control [14], [15], [16], while including FDG-positive lymph nodes in the gross tumor volume has been associated with improved regional control and survival [17]. Together, these data support FDG-PET–guided de-escalation of elective radiotherapy.

However, by reducing the radiation dose and leaving elective target volumes unchanged two randomized phase III trials did not meet their primary toxicity endpoints (normalcy of diet and dysphagia at one year) while suggesting less acute toxicity [16], [18]. Whether FDG-PET-informed volumetric de-escalation reduces toxicity, to our knowledge, has neither been assessed head to head in a clinical trial nor using validated NTCP modeling.

We therefore assessed, in a contemporary single-institution cohort, how reducing ENI and adopting involved-node approaches based on FDG-PET with photons, protons or bimodal therapy affect modeled risks of late dysphagia, xerostomia, and feeding-tube dependence; and whether tumor subsite or nodal spread modify these effects. By quantifying the potential toxicity benefit of these de-escalation strategies, this study aims to provide a rationale for future randomized trials investigating volumetric reduction of elective nodal irradiation.

## Methods

### Patient cohort

We conducted a retrospective plan-comparison study of 26 consecutive patients with squamous cell carcinoma of the head and neck who underwent pretreatment FDG-PET/CT and definitive radio(chemo)therapy with photon IMRT and a simultaneous integrated boost at our institution between 2021 and 2024. The most common primary tumor site was the oropharynx (OPC) with 14 cases, followed by the larynx (LC) with 5, hypopharynx (HPC) with 5, and oral cavity (OCC) with 2 cases. Tumor stage was scored according to the 7th edition of AJCC/UICC to harmonize nodal staging between HPV-related and −unrelated cancers. Nodal stages were N0 in 4 patients, N1 in 5, N2 in 15, and N3 in 2. Thirteen patients had unilateral (N1-2b), and 9 had bilateral lymph node metastases (all N2c-3). Twenty-three patients received concurrent chemotherapy. Treatment-associated toxicity (CTCAE version 5.0), feeding tube dependency, and body weight were extracted from clinical records. This study was approved by the ethics committee of the Medical Faculty of Heidelberg University.

### Target delineation and dose prescription

**Fig. 1:**
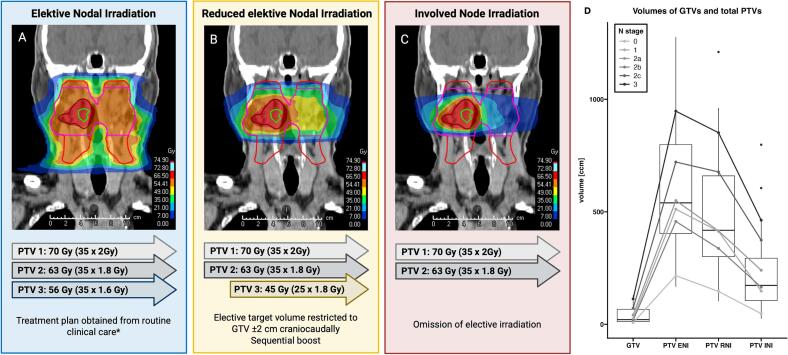
FDG-based de-escalation strategies. (A) Exemplary treatment plan of Elective Nodal Irradiation (ENI) in a patient with cT2 cN0 oropharyngeal cancer. De-escalated treatment plan of (B) Reduced Elective Nodal (RNI) and (C) Involved Node Irradiation (INI). Created using BioRender. (D) Gross tumor volume and total planning target volumes of ENI, RNI, and INI plans are displayed; lines indicate the overall median volume and dots the median volume grouped by N stage. *The dose-fractionation schedule presented represents the most used regimen in our cohort.

The target delineation of the gross tumor (GTV) and clinical high and intermediate-risk areas (CTV) based on FDG-PET and conventional imaging was adopted from the patients’ original treatment plan. All treatment plans included ENI to lymph node levels as recommended by international consensus [19]. For ENI, a simultaneous integrated boost up to 70.4 Gy, 64.0 Gy, and 57.6 Gy in 32–35 fractions was prescribed to the high-risk, intermediate-risk, and elective planning target volumes (PTVs) (Fig. 1A). For Reduced Elective Nodal Irradiation (RNI), the elective clinical target volume of metabolically and anatomically unsuspicious lymph node regions was restricted to 2 cm cranial and caudal of the primary tumor applied to both sides and 2 cm within lymph node metastases of the respective side of the neck (Fig. 1B). To the reduced elective PTV, 45.0 Gy was prescribed in 25 fractions, complemented by a sequential boost up to a cumulative total dose of 70 Gy and 63 Gy in 35 fractions to the high and intermediate-risk PTVs. For Involved Nodal Irradiation (INI), the elective PTV was omitted with identical dose prescription to high- and intermediate-risk PTVs (Fig. 1C). All PTVs were created with a margin of 3 mm to the respective CTVs. Dose constraints originally defined for original treatment plans were also applied to de-escalation strategies to minimize planner bias.

Organs at risk were auto-contoured using Limbus (Radformation) and MVision AI and manually adapted according to consensus criteria [20], [21]. Treatment planning was conducted using RayStation (V 2024B, Raysearch Laboratories AB).

### Modelling of NTCPs

Dose-volume histograms (DVHs) of de-escalated and original treatment plans based on the patient’s baseline CT were extracted from the planning system. Normal-tissue complication probabilities (NTCPs) were calculated using the validated models for grade ≥ 2 dysphagia by Christianen et al. [4], moderate-to-severe patient-rated xerostomia by Beetz et al. [9], and tube feeding dependency by Wopken et al. [22], all evaluated six months after radiotherapy. Thresholds for a clinically meaningful risk reduction were adopted from the Dutch model-based proton–photon selection system [23]. Additionally, NTCPs for acute oral mucositis grade 3 [24], as well as the long-term toxicities, aspiration assessed by videofluoroscopy [25], laryngeal edema grade ≥ 2 assessed by fiberoptic examination [6], trismus defined as jaw-opening < 35 mm [26], and primary hypothyroidism defined as TSH values > 4 mE/L [7] were calculated. Individual and overall NTCP differences between original and de-escalated treatment plans were determined. The NTCP models, including the co-primary endpoints, are predominantly mean-dose–driven: the dysphagia model uses mean dose to the superior pharyngeal constrictor and supraglottic larynx, the xerostomia model uses mean contralateral parotid gland dose, and the tube feeding model incorporates mean doses to multiple swallowing structures (Supplementary Table 4). Consequently, de-escalation that lowers the mean organ-at-risk dose will inherently reduce predicted toxicity risk. To maintain consistency despite some incomplete documentation of baseline symptoms within this retrospective cohort, no pre-treatment dysphagia, xerostomia, or weight loss was assumed for NTCP modelling.

### Statistical analysis

Statistical analyses were conducted using R (v4.4.1). Validated NTCPs for dysphagia, tube feeding, and xerostomia were prespecified as co-primary endpoints. For each NTCP, within-patient differences between ENI and de-escalated plans were tested using one-sample t-tests.

Multiplicity was handled via a two-tier serial gatekeeping procedure. Tier 1 tested INI vs ENI across dysphagia, tube feeding, and xerostomia using the Hochberg step-up method (α = 0.05). Tier 2 tested RNI vs ENI only for endpoints significant at Tier 1; if multiple endpoints entered Tier 2, we again applied Hochberg at α = 0.05 across those tests. Adjusted p-values are reported.

An exploratory effect-modification analysis was performed by comparing the within-patient differences between ENI and de-escalated plans between subgroups stratified by nodal status or primary site using the Welch two-sample *t*-test. The effect of de-escalation on secondary NTCPs was assessed exploratory using a *t*-test on within-patient differences as described above. For secondary NTCPs, we controlled multiplicity within each de-escalation strategy, and for effect modification, we controlled multiplicity within each de-escalation strategy and NTCP using Benjamini–Hochberg FDR (q = 0.05), respectively.

Absolute volume, dose, and NTCPs of the different treatment strategies are described using median and interquartile distance or range. The paired difference of these metrics between standard and de-escalated plans is described as the mean reduction (positive values indicate sparing), standard deviation and, for NTCPs, 95%-confidence intervals.

## Results

### Clinical outcomes of standard elective nodal irradiation

Twenty-six patients with squamous cell carcinoma who underwent pretreatment FDG-PET/CT were included. Baseline patient characteristics are summarized in Table 1. During treatment, 9 patients received tube feeding or parenteral nutrition. Of 16 patients with complete weight data, 12 experienced severe weight loss. Within six months after radiotherapy, nine patients had grade ≥ 2 xerostomia, and five had grade ≥ 2 dysphagia (Supplementary Table 1). After a median follow‑up of 15 months, one local, one locoregional, and one regional recurrence occurred in high-dose volumes. One patient developed distant metastasis. There was no failure in elective nodal volumes.

**Table 1:** Baseline patient characteristics and tumor stage according to the 7th edition of the AJCC/UICC TNM classification.

*Patient characteristics*	*Median*	Range
Age (years)	67	34 – 89
*Patient characteristics*	*no. (n = 26)*	%
Male	21	81
ECOG		
0	14	54
1	11	42
NA	1	4

*Disease characteristics*	*no. (n = 26)*	%

Primary site		
Oral cavity	2	8
Oropharynx	14	54
Hypopharynx	5	19
Larynx	5	19
T-stage		
1	2	8
2	10	38
3	7	27
4a	7	27
N-stage		
0	4	15
1	5	19
2a	1	4
2b	7	27
2c	7	27
3	2	8

### Dosimetry benefits of elective neck de-escalation

FDG-PET adaptive de-escalated radiotherapy of the elective neck substantially reduces target volumes. Median total PTV was 540 cm^3^ (IQR 404 to 799 cm^3^) with ENI, 418 cm^3^ (301 to 660 cm^3^) with RNI, and 173 cm^3^ (105 to 294 cm^3^) with INI (Fig. 1D).

**Fig. 2:**
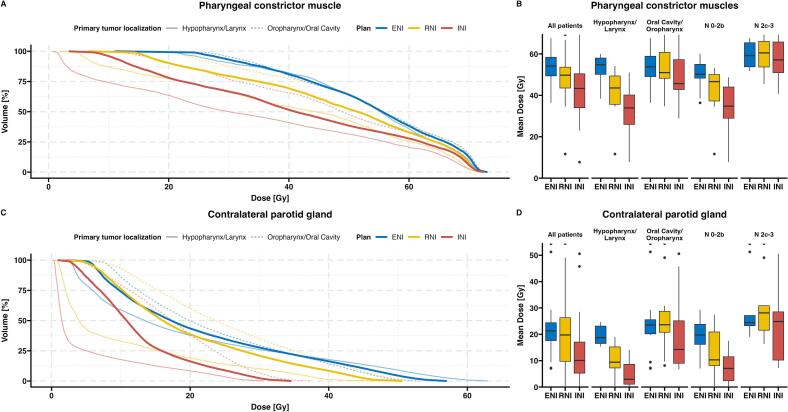
Dose sparing effect of elective neck de-escalation. Averaged DVH and mean dose (A and B) at the pharyngeal constrictor muscles (PCM) and (C and D) the contralateral parotid gland. Mean dose boxplots show entire cohort, grouping by primary tumor, and N stage; lines indicate median and interquartile range.

De-escalation of the neck spares critical organs at risk. In the entire cohort, the pharyngeal constrictor (PCM, composite) received a median D_mean_ of 54.2 Gy with ENI (IQR 49.4 to 58.4 Gy), whereas RNI and INI reduced PCM D_mean_ by a mean of 5.3 Gy (SD 8.0 Gy), and 11.0 Gy (SD 10.4 Gy) (Fig. 2A, B). At the contralateral parotid gland (cPG) ENI resulted in a D_mean_ of 21.3 Gy (IQR 17.6 to 24.4 Gy), which RNI reduced by 2.9 Gy (SD 6.9 Gy) and INI by 8.9 Gy (SD 8.3 Gy) (Fig. 2C, D).

However, dose-sparing depended on primary site and nodal stage. In OPC/OCC, RNI altered organs at risk dose only slightly (PCM D_mean_ reduction 1.4 Gy, SD 4.5; cPG −1.2 Gy, SD 3.4), whereas INI produced substantial reductions (PCM 5.7 Gy, SD 7.0; cPG 5.2 Gy, SD 7.8) (Fig. 2B, D). In N2c–N3 disease, neither de-escalation strategy showed a consistent effect (RNI: PCM 1.1 Gy, SD 5.5; cPG −0.64 Gy, SD 4.8; INI: PCM 3.7 Gy, SD 6.6; cPG 4.7 Gy, SD 7.1). A breakdown by no, unilateral, and bilateral nodal disease is provided in Supplementary Fig. 2. Localization of the primary tumor and nodal spread determined dosimetry benefits.

### Proton therapy enables further organs at risk sparing

**Fig. 3:**
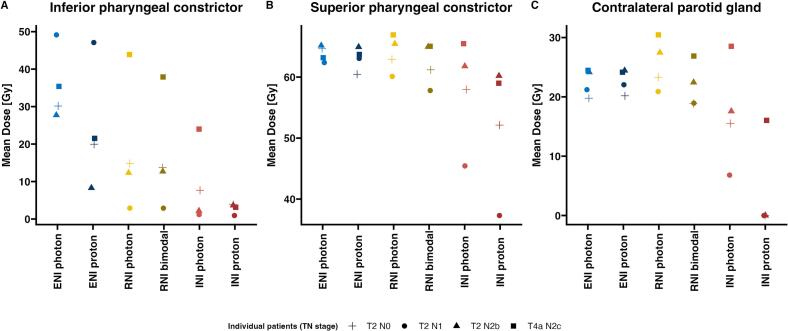
Standard proton ENI, de-escalated proton bimodal RNI and proton only INI compared to standard and de-escalated photon plans regarding the mean dose at (A) the inferior pharyngeal constrictor, (B) the superior pharyngeal constrictor, and (C) the contralateral parotid gland, in four representative oropharyngeal cancer patients.

As elective neck de-escalation achieved only moderate dosimetry benefits in oropharyngeal primaries, the potential of proton therapy to optimize organs at risk sparing was assessed in four representative OPC patients. Proton therapy with standard elective volumes substantially reduced D_mean_ at the inferior pharyngeal constrictor (iPCM) by a median of 12.0 Gy RBE (range 2.0 to 19.4) but failed to spare the superior pharyngeal constrictor (sPCM; −0.2 Gy RBE, −0.7 to 4.2) or the cPG (−0.3 Gy RBE, −0.8 to 0.3) (Fig. 3). Photon therapy with de-escalation of the elective neck also reduced iPCM dose. Compared to photons, de-escalated proton radiotherapy further improved iPCM sparing in only one patient with T4a N2c disease (RNI: 0.6 Gy RBE, −0.4 to 6.0; INI: 2.0 Gy RBE, −1.5 to 20.9) (Fig. 3A). Bimodal RNI, combining a proton boost and a photon base plan, reduced the D_mean_ at the sPCM by 1.8 Gy RBE (0.4 to 2.3) and 4.0 Gy RBE at the cPG (1.9 to 5.0) (Fig. 3B, C). Proton therapy had the greatest effect when applied with INI volumes, reducing D_mean_ by 6.2 Gy RBE at the sPCM (1.6 to 8.1) and 14.0 Gy RBE at the cPG (6.8 to 17.6) (Fig. 3B, C). In selected cases, de-escalated proton radiotherapy spared organs at risk that could not be spared by de-escalating photon radiotherapy alone.

### Reduced elective and involved-node irradiation mitigate modeled toxicity risk

**Fig. 4:**
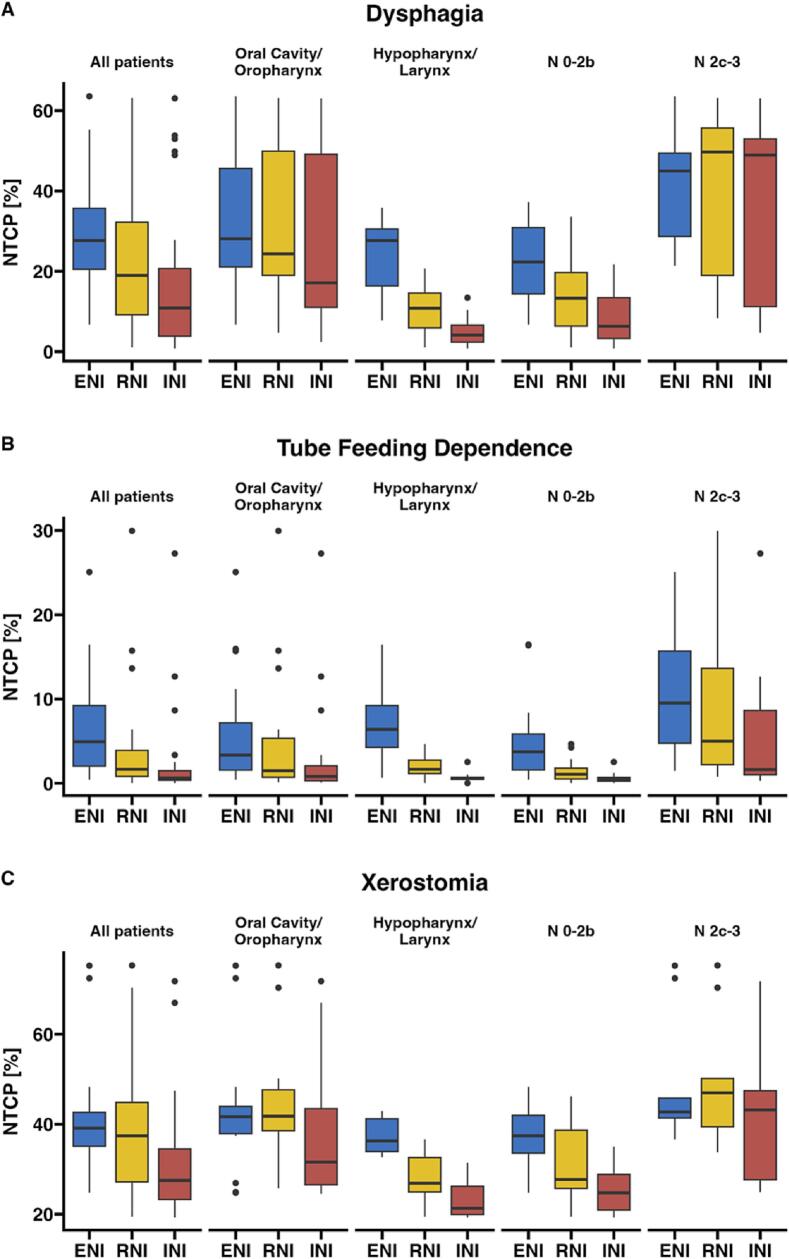
De-escalation of the elective neck reduces modeled toxicity risk. NTCP of (A) dysphagia, (B) tube feeding dependence, and (C) xerostomia in standard and de-escalated plans are displayed in boxplots for all patients, grouped by primary tumor, and N stage.

Next, we assessed whether the dosimetric advantages translated into lower model-predicted risks of radiotherapy-associated toxicities. Photon RNI and INI significantly lowered the predicted risks of dysphagia by a mean of 5.9 pp (95% CI 2.3 to 9.6 pp; p_adj_ = 0.005) and 11.0 pp (6.9 to 15.2 pp; p_adj_ < 0.001), and tube feeding dependence by 2.8 pp (1.2 to 4.5 pp; p_adj_ = 0.005) and 4.2 pp (2.5 to 6.0 pp; p_adj_ < 0.001) compared to ENI (Fig. 4A, B). RNI-mediated reduction of modeled xerostomia risk by 2.8 pp (0.0 to 5.7 pp; p_adj_ = 0.054) was not statistically significant but INI reduced modeled xerostomia risk by 8.8 pp (5.5 to 12.2 pp; p_adj_ < 0.001) (Fig. 4C).

The extent of NTCP reduction achieved through de-escalation varied depending on primary tumor and nodal stage. In HPC/LC patients, the mean dysphagia risk reduction by RNI and INI was 13.4 and 18.4 pp whereas OPC/OCC patients experienced significantly lower risk reductions by 1.3 pp (q = 0.002) and 6.3 pp (q = 0.009). Similarly, in patients with N0-2b compared to N2c-3 disease, dysphagia risk reduction appeared greater for both RNI (7.9 vs. 2.3 pp; q = 0.136) and INI (13.9 vs. 5.6 pp; q = 0.054). In contrast to stratification by primary tumor, nodal subgroup differences did not meet significance, adjusted for multiple testing (Supplementary Table 2).

**Fig. 5:**
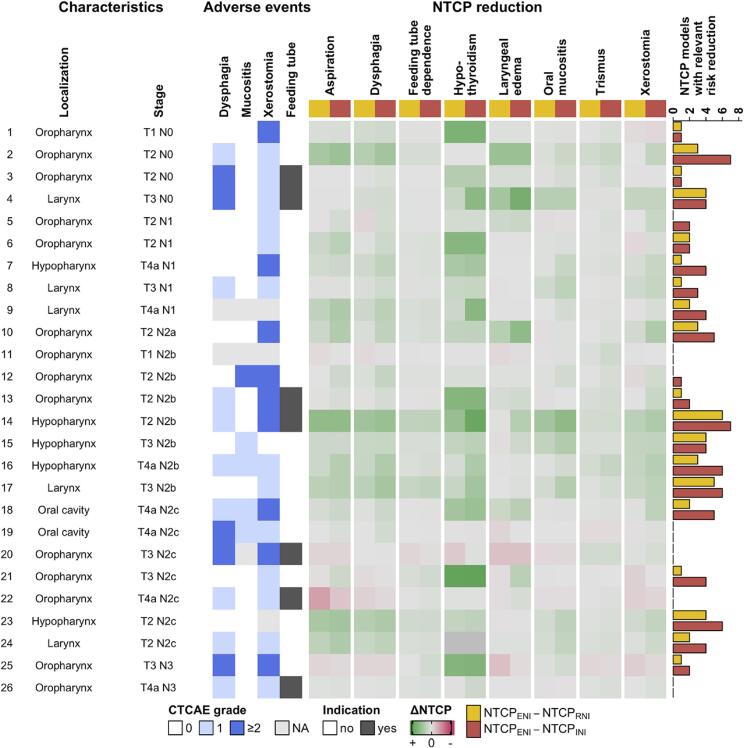
Individual NTCP reductions with de-escalated radiotherapy. Patient characteristics and observed adverse events are displayed on the left. The central heatmap shows NTCP reduction of RNI (upper row, yellow) and INI (lower row, red) compared to ENI for aspiration, dysphagia, tube feeding dependence, hypothyroidism, laryngeal edema, oral mucositis, trismus, and xerostomia; green shading indicates NTCP reduction, red shading indicates NTCP increase. Bars on the right indicate the number of NTCPs with clinically relevant risk reduction (≥ 10%, or ≥ 5% for tube feeding) for RNI and INI per patient. (For interpretation of the references to colour in this figure legend, the reader is referred to the web version of this article.)

Overall, using the criteria of the Dutch model-based proton–photon selection system, 9 of 26 patients using RNI and 18 of 26 patients using INI would have achieved a clinically meaningful risk reduction for dysphagia, tube feeding dependence, and/or xerostomia. Regarding other common radiotherapy-associated toxicities, the greatest mean NTCP reductions were observed in hypothyroidism, 17.9 pp in RNI (95% CI 10.3 to 25.4 pp) and 24.1 pp in INI (16.1 to 32.0 pp), aspiration, 6.3 pp in RNI (1.2 to 11.4 pp) and 10.9 pp in INI (5.9 to 15.9 pp), and oral mucositis, 3.7 pp in RNI (0.7 to 6.7 pp) and 8.1 pp in INI (4.1 to 12.1 pp) (Fig. 5; Supplementary Table 3). Considering all eight evaluated toxicity models at least one clinically relevant risk reduction was projected for 19 of 26 patients with RNI and 21 of 26 patients with INI. Per patient, a median of 1 clinically relevant risk reduction was predicted for RNI (IQR 0.25 to 3), and 3.5 (1 to 4.75) for INI (Fig. 5). Thus, according to NTCP models, de-escalated neck radiotherapy is predicted to mitigate clinically relevant radiotherapy-associated late toxicities.

## Discussion

This within-patient plan-comparison suggests the potential of FDG-PET/CT–guided elective radiotherapy de-escalation to mitigate modeled risk of late toxicities in the head and neck region. De-escalation reduced the total planning target volume and lowered organ at risk dose. Both de-escalation strategies, RNI and INI, significantly decreased the modeled risk of dysphagia and feeding-tube dependence. Predicted xerostomia risk was reduced by INI but not by RNI. The de-escalation benefit was more pronounced in hypopharyngeal and laryngeal than in oropharyngeal and oral cavity carcinomas. In an exploratory subset of oropharyngeal carcinoma patients, de-escalated proton therapy enhanced dose sparing beyond both standard elective neck proton and de-escalated photon radiotherapy.

The limited benefit of RNI in oropharyngeal and oral cavity carcinoma is anatomically plausible. A 2-cm elective margin to the primary and involved nodes leaves the pharyngeal constrictor and contralateral parotid gland adjacent to elective regions, curtailing the reduction of modeled toxicity risk. Similarly, bilateral lymph node metastases permit only moderate elective target volume reduction and tend to impair risk reduction compared to no or unilateral nodal disease. Despite heterogeneity by primary site and nodal metastases, reducing elective nodal irradiation is predicted to confer a clinically meaningful reduction of at least one toxicity risk for most patients.

Toxicity reduction by de-escalation of head and neck radiotherapy is subject to ongoing clinical investigation. Two randomized trials that reduced the elective-neck dose without altering elective volumes improved acute dysphagia and feeding-tube outcomes yet missed their prespecified late swallowing endpoints [16], [18]. Thus, lowering the elective dose alone might be insufficient to modify late toxicity. This plan-comparison study shows that volumetric FDG-PET–guided de-escalation mitigates the modeled dysphagia risk 6 months after radiotherapy. Consistent with the volumetric de-escalation in our INI plans, the single-arm INRT-AIR trial reported preserved swallowing function and no quality-of-life deterioration after involved-node–only irradiation [15]. By providing effect estimates for two de-escalation strategies and information on effect modification by primary site and laterality of lymph node metastases, these results may aid the design of future randomized trials.

A recently published randomized phase III trial comparing proton with photon radiotherapy for oropharyngeal carcinoma demonstrated a significant reduction in gastrostomy tube placement with proton therapy [27]. Our exploratory proton data align with these findings: in representative oropharyngeal cases, proton-based ENI reduced dose to the lower swallowing apparatus compared to photon ENI. Notably, the benefit of protons appeared most pronounced when combined with elective nodal de-escalation. In INI proton plans, mean doses to the pharyngeal constrictors and contralateral parotid gland approached near-complete sparing in select patients—a degree of organ-at-risk protection unattainable with photon de-escalation or standard proton treatment alone. However, proton therapy resources are limited, and patient selection is essential. Our exploratory subset of four oropharyngeal patients is too small to identify which clinical characteristics, such as primary tumor subsite, T-stage, or nodal status, are associated with a predicted reduction in adverse events through proton therapy. The substantial interindividual variation in organ-at-risk doses observed across treatment strategies suggests that NTCP-based selection frameworks [23] could be used to identify patients for whom proton therapy provides meaningful additional sparing beyond photon de-escalation alone. Prospective investigation of combined volumetric de-escalation and proton therapy is warranted.

Elective neck de-escalation could be oncologically safe in selected patients. In this cohort, no isolated elective nodal failures occurred after standard ENI. Recent pattern-of-failure analyses attribute most recurrences to high-dose rather than low-dose volumes [28], [29], and elective coverage limited to involved nodes or levels adjacent to gross disease appears to retain low recurrence rates [15], [30], [31]. However, systematic analysis of larger cohorts is needed to quantify failure risk in de-escalated volumes.

Prior retrospective comparisons have hinted that de-escalation reduces dose to lymphocyte-rich structures, and, thereby, might mitigate lymphopenia and improve survival [15], [32], [33]. Radiation-induced lymphopenia is associated with worse outcomes in head and neck carcinoma [34], but whether elective neck de-escalation mitigates lymphopenia warrants further investigation.

This study delivers, to our knowledge, the first within-patient comparison of three elective-neck strategies—ENI, RNI, and INI—using identical high- and intermediate-risk target definitions and dose constraints to minimize planner bias. We probe effect heterogeneity by subsite and nodal stage, assess clinical relevance against predefined NTCP thresholds, and add exploratory proton plans to test additional sparing when photon de-escalation plateaus. Together, the paired design and validated NTCP modeling provide a clinically informative assessment of contemporary elective neck de-escalation strategies.

The principal limitation of this study is its retrospective, single-institution, *in silico* plan-comparison design. Subgroup analyses are limited in statistical power. The proton plan comparison is exploratory. Finally, incomplete documentation of baseline symptoms required assuming no pre-treatment toxicity, partially underestimating absolute toxicity risk.

Together, these data support FDG-PET/CT–guided volumetric de-escalation, with INI—and, to a lesser extent, RNI—as strong candidates to reduce swallowing-related toxicity, pending clinical validation. Patients with hypopharyngeal or laryngeal carcinoma are predicted to benefit most from elective radiotherapy de-escalation. Proton therapy may be considered in selected cases in which photon-based de-escalation is insufficient to spare the pharyngeal constrictors or the contralateral parotid gland. These data support prospective evaluation of volumetric de-escalation, with the effect estimates reported here informing trial design and patient selection.

## Data Availability

The datasets generated and analyzed during the current study are available from the corresponding author on reasonable request.

## Declaration of Generative AI and AI-assisted technologies in the writing process

After drafting this manuscript, the authors used ChatGPT 5 to improve readability and language. After using this tool, the authors reviewed and edited the content as needed and take full responsibility for the content of the publication.

## Funding

This work was supported by a Clinician-Scientist scholarship from the Faculty of Medicine, Heidelberg University to Florian Stritzke.

## Declaration of competing interest

The authors declare that they have no known competing financial interests or personal relationships that could have appeared to influence the work reported in this paper.
