# Supplementary material for: De-escalation of elective radiotherapy guided by FDG-PET lowers modeled late swallowing-related toxicity in head and neck cancer
Source: Clin Transl Radiat Oncol. 2026 Apr 1;59:101156. doi: 10.1016/j.ctro.2026.101156 (PMC13092707; doi:10.1016/j.ctro.2026.101156)
Supplement: Supplementary Data 1 [file mmc1.docx]

Supplementary Table 1.

| *Toxicity* | *no. (n=26)* | % |
| --- | --- | --- |
| Xerostomia (CTCAE) |  |  |
| 0 | 1 | 4 |
| 1 | 13 | 50 |
| 2 | 9 | 35 |
| NA | 3 | 12 |
| Dysphagia (CTCAE) |  |  |
| 0 | 10 | 38 |
| 1 | 9 | 35 |
| 2 | 4 | 15 |
| 3 | 1 | 4 |
| NA | 2 | 8 |
| Nutritional support during therapy |  |  |
| PEG feeding tube | 5 | 19 |
| Parenteral nutrition | 4 | 15 |
| Denied | 2 | 8 |
| None | 15 | 62 |
| Weight loss during therapy/at first follow up |  |  |
| Moderate (1-10% of body weight) | 2 | 8 |
| Severe (>10% of body weight) | 12 | 46 |
| None | 2 | 8 |
| NA | 10 | 38 |

Supplementary Table 1. Maximum grade of toxicity according to CTCAE version 5 and nutritional status during follow-up visits within six months.

Supplementary Table 2

| ***NTCP*** | ***De-escalation*** | ***OPC/OCC (n)*** | ***OPC/OCC (mean ± SD)*** | ***HPC/LC (n)*** | ***HPC/LC (mean ± SD)*** | ***p*** | ***FDR (BH)*** |
| --- | --- | --- | --- | --- | --- | --- | --- |
| Dysphagia | INI vs ENI | 16 | 6.3 ± 8.5 | 10 | 18.6 ± 8.7 | 0.002 | 0.005 |
|  | RNI vs ENI | 16 | 1.3 ± 6.4 | 10 | 13.4 ± 7.6 | <0.001 | 0.001 |
| Tube Feeding | INI vs ENI | 16 | 2.5 ± 2.7 | 10 | 6.9 ± 5.2 | 0.031 | 0.062 |
|  | RNI vs ENI | 16 | 1.1 ± 2.3 | 10 | 5.6 ± 4.8 | 0.018 | 0.037 |
| Xerostomia | INI vs ENI | 16 | 5.4 ± 8.2 | 10 | 14.2 ± 5.2 | 0.003 | 0.005 |
|  | RNI vs ENI | 16 | -1.4 ± 3.9 | 10 | 9.5 ± 5.7 | <0.001 | <0.001 |
| ***NTCP*** | ***De-escalation*** | ***N0-2b (n)*** | ***N0-2b (mean ± SD)*** | ***N2c-3 (n)*** | ***N2c-3 (mean ± SD)*** | ***p*** | ***FDR (BH)*** |
| Dysphagia | INI vs ENI | 17 | 13.9 ± 9.7 | 9 | 5.6 ± 9.7 | 0.054 | 0.054 |
|  | RNI vs ENI | 17 | 7.9 ± 8.9 | 9 | 2.3 ± 8.5 | 0.136 | 0.136 |
| Tube Feeding | INI vs ENI | 17 | 4.4 ± 4.7 | 9 | 3.9 ± 3.7 | 0.774 | 0.774 |
|  | RNI vs ENI | 17 | 3.5 ± 4.2 | 9 | 1.6 ± 3.7 | 0.253 | 0.253 |
| Xerostomia | INI vs ENI | 17 | 11.0 ± 8.0 | 9 | 4.7 ± 7.5 | 0.062 | 0.062 |
|  | RNI vs ENI | 17 | 4.8 ± 7.2 | 9 | -0.9 ± 5.4 | 0.036 | 0.036 |

Supplementary Table 2. Subgroup differences in NTCP reductions. Primary tumor and nodal stage subgroups are compared using the Welch t-test. The Benjamini–Hochberg method controlled the False Discovery Rate (FDR) per NTCP × De-escalation.

Supplementary Table 3

| **NTCP** | **NTCP ENI [%] (mean ± SD)** | **Δ NTCP RNI [pp] (mean ± SD)** | **ENI vs RNI (p value)** | **ENI vs RNI (FDR-adj p)** | **Δ NTCP RNI [%] (95% CI)** | **Δ NTCP INI [pp] (mean ± SD)** | **ENI vs INI (p value)** | **ENI vs INI (FDR-adj p)** | **Δ NTCP INI [%] (95% CI)** |
| --- | --- | --- | --- | --- | --- | --- | --- | --- | --- |
| **Mucositis** | 40.0 ± 10.3 | 3.7 ± 7.4 | 0.017 | 0.022 | (0.7 – 6.7) | 8.1 ± 9.9 | <0.001 | <0.001 | (4.1 – 12.1) |
| **Aspiration** | 23.1 ± 18.8 | 6.3 ± 12.6 | 0.018 | 0.022 | (1.2 – 11.4) | 10.9 ± 12.4 | <0.001 | <0.001 | (5.9 – 15.9) |
| **Laryngeal Edema** | 57.6 ± 37.1 | 3.0 ± 10.5 | 0.160 | 0.160 | (-1.3 – 7.2) | 8.3 ± 14.4 | 0.007 | 0.007 | (2.5 – 14.1) |
| **Trismus** | 16.2 ± 7.3 | 2.2 ± 2.8 | <0.001 | 0.002 | (1.0 – 3.3) | 4.4 ± 3.7 | <0.001 | <0.001 | (2.9 – 5.9) |
| **Hypo-thyroidism** | 41.4 ± 31.4 | 17.9 ± 18.3 | <0.001 | <0.001 | (10.3 – 25.4) | 24.1 ± 19.3 | <0.001 | <0.001 | (16.1 – 32.0) |

Supplementary Table 3. Modeled risk of other toxicities. NTCP-modeled risk for mucositis, aspiration, laryngeal edema, trismus, and hypothyroidism of standard ENI plans and within-patient differences of de-escalated RNI and INI plans (Δ = ENI − de-escalated plan) are reported. Summaries are mean ± SD; tests are one-sample t-tests on Δ against 0. 95% CIs are for mean Δ from one-sample t-test. The Benjamini–Hochberg method controlled the False Discovery Rate (FDR) separately for INI vs ENI and RNI vs ENI p-values.

**Supplementary Table 4.** NTCP models applied in this study.

| Toxicity *(Reference)* | Endpoint | Model | Parameters |
| --- | --- | --- | --- |
| Oral mucositis  *(Bhide et al., 2012)* | Acute oral mucositis grade 3, during or within 8 weeks after RT | $NTCP=\frac{1}{(1+{(\frac{MD50}{D})}^{k}}$ | MD50 = 51Gy  D = mean dose to the oral mucosa (2Gy equivalent (α/β=10), converted using Withers formula); oral cavity mean dose was used as surrogate for oral mucosa  k = 1 |
| Trismus  *(Lindblom et al., 2014)* | Jaw opening <35mm | $NTCP=\frac{1}{(1+e^{4\gamma{}^{(1-\frac{D}{D_{50}})}})}$ | γ = 0.78  D = physical mean dose to ipsilateral masseter  D50 = 57.2 Gy |
| Xerostomia  *(Beetz et al., 2012)* | Moderate-to-severe patient-rated xerostomia at 6 months | $NTCP={(1+e^{-S})}^{-1}$  S = –1.443 + (mean dose contralateral parotid gland ∙ 0.047) + (baseline xerostomia score ∙ 0.720) | Baseline xerostomia score: set to 0 for all patients due to incomplete baseline data  mean dose contralateral parotid gland = physical mean dose |
| Aspiration  *(Eisbruch et al., 2011)* | Increase in videofluoroscopy-detected aspiration ≥ 12 months post-therapy compared with baseline | $NTCP=\Phi(\frac{D-{TD}_{50}}{m\cdot{TD}_{50}})$ | D = mean physical dose to the pharyngeal constrictor muscles  TD50 = 63Gy  m = 0.16 |
| Hypothyroidism *(Boomsma et al., 2012)* | Elevated TSH with normal or decreased free T4 at any point during a 2-year follow-up | $NTCP={(1+e^{-S})}^{-1}$  S = 0.011 + (0.062 ⋅ mean dose thyroid gland) + (–0.19 ⋅ thyroid gland volume) | Mean dose thyroid gland = physical mean dose |
| Tube feeding dependence  *(Wopken et al., 2014)* | Tube feeding dependence at 6 months after treatment | $NTCP={(1+e^{-S})}^{-1}$  S = ­–11.70 + (advanced T-stage ⋅ 0.43) + (moderate weight loss ⋅ 0.95) + (severe weight loss ⋅ 1.63) + (accelerated radiotherapy ⋅ 1.20) + (chemoradiation ⋅ 1.91) + (radiotherapy plus cetuximab ⋅ 0.56) + (mean dose PCM superior ⋅ 0.071) + (mean dose PCM inferior ⋅ 0.034) + (mean dose contralateral parotid ⋅ 0.006) + (mean dose cricopharyngeal muscle ⋅ 0.023) | Advanced = T3-4  Moderate / severe weight loss = set to 0 for all patients due to incomplete baseline data  physical mean doses |
| Laryngeal edema  (Rancati et al., 2009) | Grade ≥2 laryngeal edema within 15 months after radiotherapy | $NTCP=\frac{1}{1+{(\frac{D_{50}}{D})}^{k}}$ | Logit model applied to mean laryngeal dose as a simplification of the original EUD-based model  D50 = 47Gy  D = mean dose to the larynx (2Gy/fr equivalent (α/β=3 Gy), converted using Withers formula)  k = 7.33 |
| Dysphagia  (Christianen et al., 2012) | Physician-rated swallowing dysfunction at 6 months | $NTCP={(1+e^{-S})}^{-1}$  S = –6.09 + (0.057 ⋅ mean dose superior PCM) + (0.037 ⋅ mean dose supraglottic larynx) | Physical mean doses for superior PCM and larynx |

For each toxicity endpoint, the model formula, key input variables, parameter values, and assumptions are listed.
