# Supplementary figures and images for: De-escalation of elective radiotherapy guided by FDG-PET lowers modeled late swallowing-related toxicity in head and neck cancer
Source: Clin Transl Radiat Oncol. 2026 Apr 1;59:101156. doi: 10.1016/j.ctro.2026.101156 (PMC13092707; doi:10.1016/j.ctro.2026.101156)

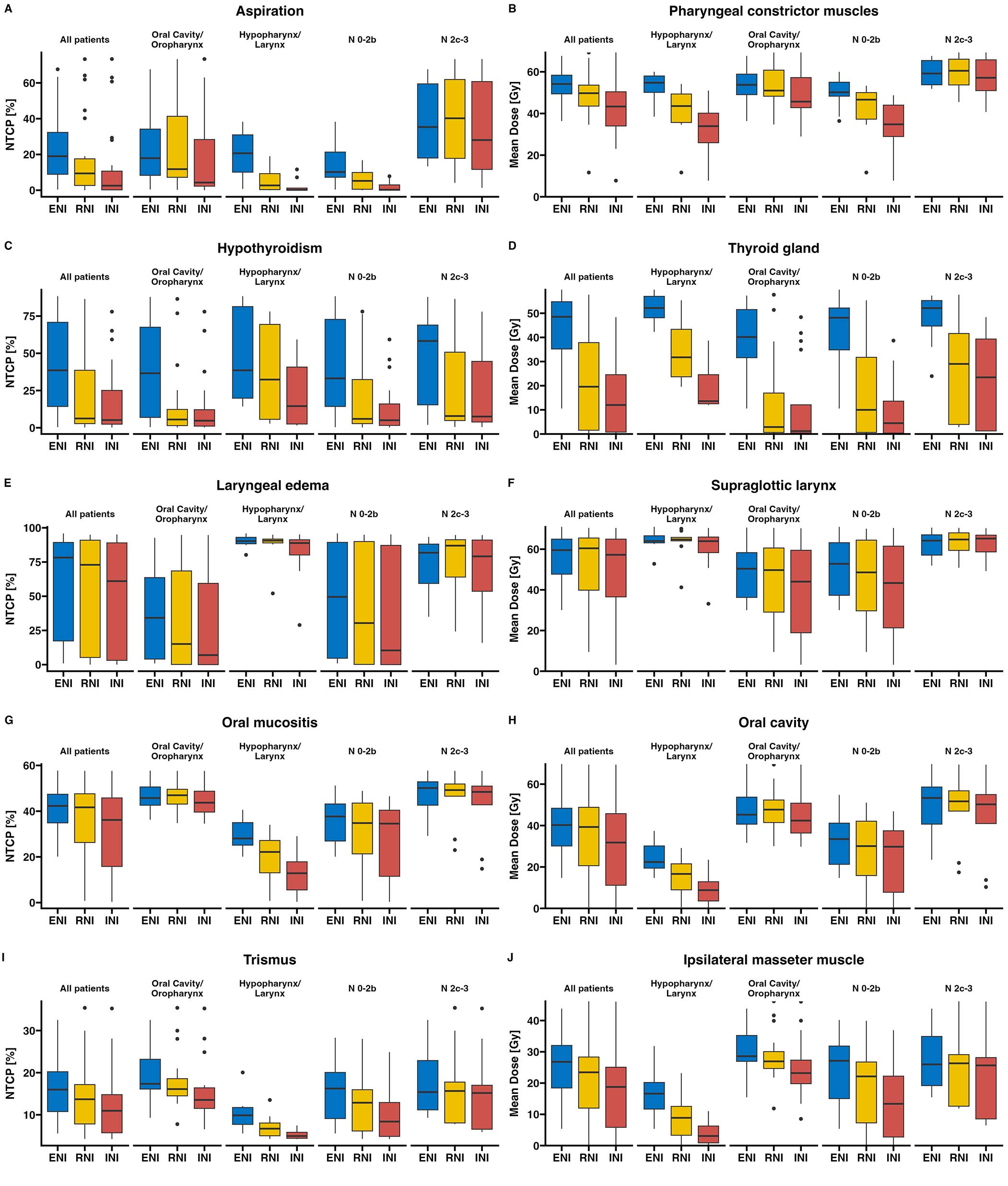

Supplement: Supplementary Figure 2 [file mmc2.jpg]

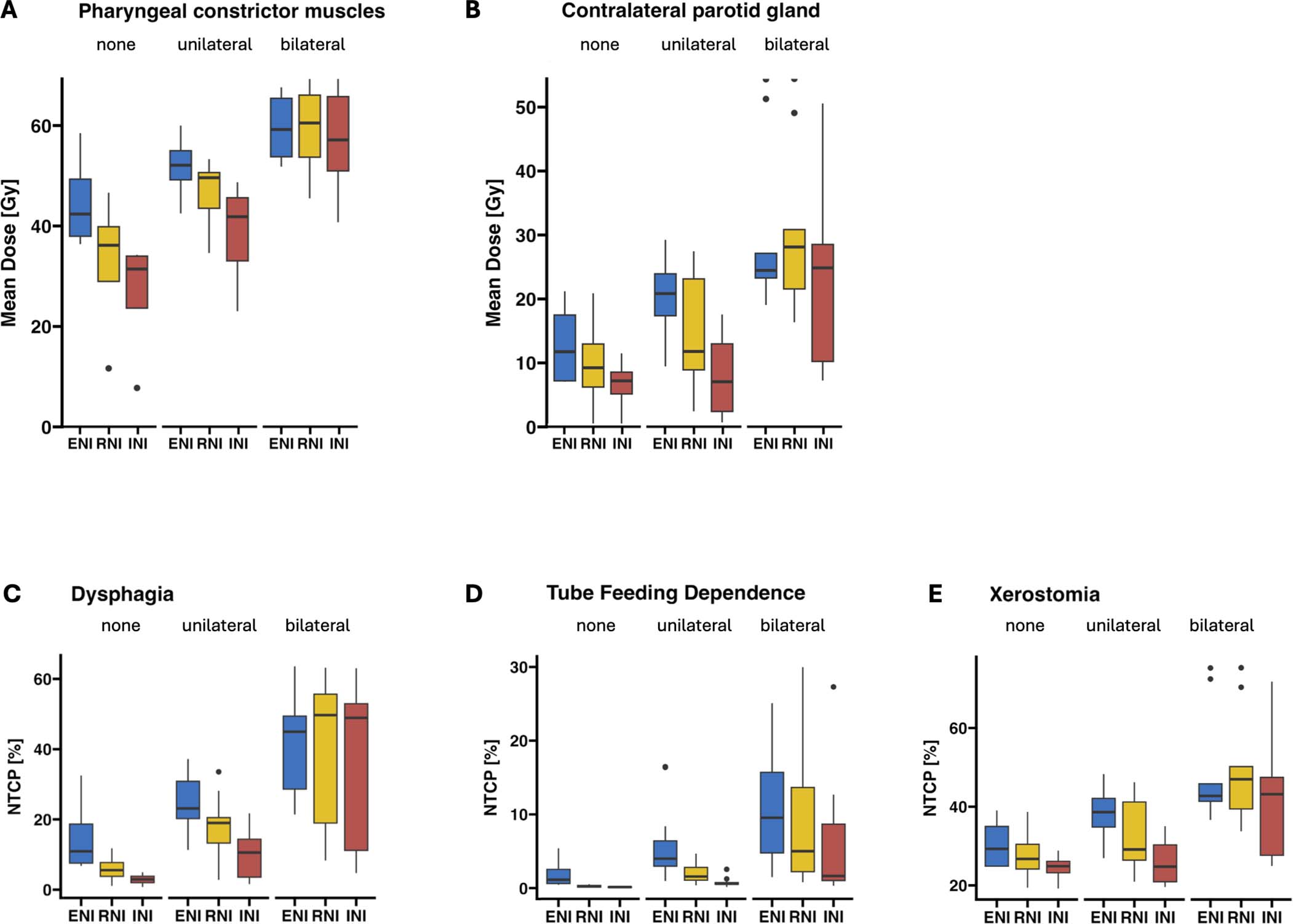

Supplement: Supplementary Figure 3 [file mmc3.jpg]
